# The Restorative Effect of Human Amniotic Fluid Stem Cells on Spinal Cord Injury

**DOI:** 10.3390/cells10102565

**Published:** 2021-09-28

**Authors:** Maryam Lale Ataei, Mohammad Karimipour, Parviz Shahabi, Roghiyeh Pashaei-Asl, Esmaeil Ebrahimie, Maryam Pashaiasl

**Affiliations:** 1 Neuroscience Research Center, Tabriz University of Medical Sciences, Tabriz 5166614766, Iran; Lalataeem@tbzmed.ac.ir; 2 Department of Anatomical Sciences, Faculty of Medicine, Tabriz University of Medical Sciences, Tabriz 5166614766, Iran; Karimipourm@tbzmed.ac.ir; 3 Drug Applied Research Center, Tabriz University of Medical Sciences, Tabriz 5166614766, Iran; Shahabip@tbzmed.ac.ir; 4 Department of Physiology, Faculty of Medicine, Tabriz University of Medical Sciences, Tabriz 5166614766, Iran; 5 Department of Clinical Biochemistry, Faculty of Medicine, Tehran University of Medical Sciences, Tehran 1417653911, Iran; rogayepashaei@gmail.com; 6 School of Life Sciences, College of Science, Health and Engineering, La Trobe University, Melbourne, VIC 3086, Australia; E.Ebrahimie@latrobe.edu.au; 7 Genomics Research Platform, Research & Industry Engagement, La Trobe University, Melbourne, VIC 3086, Australia; 8 School of Animal and Veterinary Sciences, The University of Adelaide, Adelaide, SA 5371, Australia; 9 School of BioSciences, Faculty of Science, The University of Melbourne, Melbourne, VIC 3010, Australia; 10 Stem Cell Research Center, Tabriz University of Medical Sciences, Tabriz 5166614766, Iran; 11 Department of Reproductive Biology, Faculty of Advanced Medical Science, Tabriz University of Medical Science, Tabriz 5166614766, Iran; 12 Women’s Reproductive Health Research Center, Tabriz University of Medical Sciences, Tabriz 5166614766, Iran

**Keywords:** human amniotic fluid, mesenchymal stem cells, conditioned medium spinal cord injury, transplantation, neural regeneration

## Abstract

Spinal cord injury (SCI) is a debilitating condition within the neural system which is clinically manifested by sensory-motor dysfunction, leading, in some cases, to neural paralysis for the rest of the patient’s life. In the current study, mesenchymal stem cells (MSCs) were isolated from the human amniotic fluid, in order to study their juxtacrine and paracrine activities. Flow cytometry analysis was performed to identify the MSCs. A conditioned medium (CM) was collected to measure the level of BDNF, IL-1β, and IL-6 proteins using the ELISA assay. Following the SCI induction, MSCs and CM were injected into the lesion site, and also CM was infused intraperitoneally in the different groups. Two weeks after SCI induction, the spinal cord samples were examined to evaluate the expression of the doublecortin (*DCX*) and glial fibrillary acid protein (*GFAP*) markers using immunofluorescence staining. The MSCs’ phenotype was confirmed upon the expression and un-expression of the related CD markers. Our results show that MSCs increased the expression level of the *DCX* and decreased the level of the *GFAP* relative to the injury group (*p* < 0.001). Additionally, the CM promoted the *DCX* expression rate (*p* < 0.001) and decreased the *GFAP* expression rate (*p* < 0.01) as compared with the injury group. Noteworthily, the restorative potential of the MSCs was higher than that of the CM (*p* < 0.01). Large-scale meta-analysis of transcriptomic data highlighted *PAK5*, *ST8SIA3*, and *NRXN1* as positively coexpressed genes with *DCX*. These genes are involved in neuroactive ligand–receptor interaction. Overall, our data revealed that both therapeutic interventions could promote the regeneration and restoration of the damaged neural tissue by increasing the rate of neuroblasts and decreasing the astrocytes.

## 1. Introduction

Spinal cord injury (SCI) is a neurological trauma that leads to neural tissue degeneration and glial scar formation, which may result in permanent disability and insufficient functional recovery for the rest of the patient’s life [1,2]. Additionally, SCI is associated with severe neuropathic pain and sensory-motor disorders [3]. SCI treatment and rehabilitative interventions are costly activities that affect people in every community. One of the most considerable outcomes of SCI is the neural cell loss and glial scar formation, which should be taken into therapeutic consideration [4]. Neurogenesis that follows SCI plays a key role in the regeneration phenomena; thus, efforts to compensate for the neural cell loss are much needed [5,6]. Glial scar, as the unusual rearrangement of the hypertrophy astrocytic processes with high expression of *GFAP* (as a marker of astrocyte), is a major obstacle in the axon regeneration and the extracellular matrix produced by these cells, which is mainly comprised of chondroitin-sulfated (CS) and proteoglycans (PG) [2,3]. Unlike the adult brain, the spinal cord lacks neurogenesis ability during adulthood; therefore, finding effective therapeutic strategies for repairing and improving the affected spinal cord is very important [7,8]. In this regard, stem cells and their products have been developed as a possible and efficient strategy to help repair the SCI [9,10]. In addition to neural cell death, neuroinflammatory and immunological reactions occurring during the SCI, draws attention to the use of MSCs [8]. Moreover, MSCs can be harvested from multiple sources and in large numbers, and can be used in the case of autologous transplantation [9]. An important source for the isolation of MSCs is the human amniotic fluid, which is usually obtained from amniocentesis in the second trimester [11]. Moreover, human amniotic MSCs (hAF-MSCs) can endure continuous passages without alteration in the normal phenotype and karyotype during the cell culture [12,13]. hAF-MSCs secrete multiple growth factors (GFs), namely chemokines, exosomes, microvesicles, hormones, and interleukin (IL) in CM [14,15]. Due to it containing high amounts of molecular agents, different types of growth factors cytokines, and anti-inflammatory factors, the CM infusion seems more effective than any other factors [14,16,17]. Moreover, the preparation of CM and its administration to patients through different routes is more facile as compared with other approaches [18]. A great number of experiments showed that a variety of biological agents, including brain-derived neurotrophic factor (BDNF), interleukin 6 (IL-6), interleukin 1 beta (IL-1β), hepatocyte growth factor (HGF), and epidermal growth factor (EGF), just to name a few, exist in the MSCs-CM [19]. BDNF increases cell proliferation, neurogenesis, cell survival, and myelin synthesis, on the one hand, decreases the apoptosis, glial scar formation, and demyelination in a spinal cord injury model, on the other [19]. IL-1β and IL-6 in the CM can help axonal outgrowth and prevent neuronal death [20]. Therefore, in the present study, we aimed to explore the possible effect of the local transplantation of hAF-MSCs and injection of CM through focal and intraperitoneal (IP) routes on neurogenesis and astrocytosis in the rat model of spinal cord injury.

## 2. Materials and Methods

### 2.1. Animals and Experimental Design

In this study, adult male wistar rats (weight: 270–300 g) were purchased from the animal laboratory. The animal housing conditions were in compliance with the guidelines of the ethics committee of Tabriz University of Medical Sciences (registered number 95/5–10/7). All animals were housed under standard conditions: 12 h light/dark cycle with sufficient food and water. Forty-two adult rats were randomly assigned to seven groups (6/group): Control (laminectomy only), Injury, CM-IP, DMEM-IP, CM-Focal, DMEM-Focal, and Stem Cell. In all groups, laminectomy was performed at T9–T10 vertebral level in the dorsal surface of the spinal cord using the Infinite Horizons Impactor, with an impact force of 150 (moderate SCI) key (1 dyn = 1 g⋅cm/s^2^ = 10^−5^ kg⋅m/s^2^ = 10^−5^ N)

### 2.2. Isolation and Cultivation of MSCs from the Human Amniotic Fluid

Amniotic fluid samples of approximately 5 mL were obtained from eight mothers, who underwent amniocentesis for the routine karyotype screening in Al-Zahra hospital (Tabriz, Iran). The amniocentesis was carried out under the gynecologist’s supervision based on the sonographer guides using a 22 G Needle. Following the orientation of participants by an experienced colleague, amniotic fluid samples were donated voluntarily to this research and all of the patients filled out the consent form. After the routine test, the leftovers were dedicated to this research. The samples were transferred to the laboratory at 37 °C. First, the amniotic fluid was centrifuged at 450× *g* for 10 min at room temperature, and then the remaining pellet was washed by PBS (Gibco; Thermo Fisher Scientific, Darmstadt, Germany) until the pellet had a light color. Afterward, the cells were cultured in AmnioMAX II Complete Medium (Gibco, cat# 11269). Finally, the samples were plated into 6 well plates and incubated at 37 °C in a humidified atmosphere containing 5% CO_2_. After one week of cells being attached to the plates, the AmnioMAX II in each plate was changed two times per week. After two weeks, when the cells reached approximately 90% confluency in AmnioMAX II, they were trypsinized and neutralized. Then, the cell suspension was centrifuged and moved to T25 flasks with DMEM low glucose (DMEM-L) media in the optimized condition, where it was incubated at 37 °C in a humidified atmosphere containing 5% CO_2_.

### 2.3. MSCs Characterization and Identification

Flow cytometry analysis was performed to confirm the MSCs’ phenotype. For this purpose, cells were trypsinized, and single cells were collected into the cap filtered FACs tub. Next, pellets were washed twice using FACs solution, which were then centrifuged at 1500 RPM for 3 min. Then, the cells were incubated with CD 105 (Catalog No. 1p-298-To25 Exbio) and CD 73 (Catalog No. 561260 BD Biosciences) antibodies as mesenchymal stem cell markers and CD 45 (Catalog No. 1F-222-T025 Exbio) and CD 14 (Catalog No. 12-0149-42 eBioscience) antibodies as hematopoietic stem cell markers. Afterward, the cells were analyzed using the BD FACSCalibur system and Flow Jo software (Version 7.6.1. Cambridge, UK).

### 2.4. Preparation of MSCs-CM

For a collection of CM, initially, MSCs were cultured in T25 tissue culture flask in Dulbecco’s modified Eagle’s medium (DMEM)-low glucose, supplemented with 15% fetal bovine serum (FBS). When MSCs reached 80–90% confluence, the cells were dissociated enzymatically into single cells, which were seeded with an FBS-free DMEM-L culture medium. CM was collected from each confluent flask after 48 h of incubation with serum-free media in culture condition, centrifuged at 450× *g* for 10 min for free-floating cells removal. Then, the product was filtered through 0.22 µm filters for sterilization. Subsequently, the CM was concentrated by freeze-drying processes and stored at −80 °C for later use.

### 2.5. ELISA Assay

In order to measure the paracrine activity of the MSCs and protein level of BDNF, IL-1β, IL-6 proteins in CM, the supernatant was collected for the quantitative analysis using enzyme-linked immunosorbent assay (ELISA) kits, Human/Mouse BDNF DY248, Human IL-1β/IL-1F2 DY201, and Human IL-6 DY206 with R&D Systems DuoSet Development Kit. Briefly, 100 µL of MSC-CM was added to the ELISA. Plates were coated with a 100 µL monoclonal antibody to the factor of interest, which was incubated for 120 min at 37 °C. Having been washed with PBS, an antibody was added to the wells and the samples were incubated further for 120 min at 37 °C, which were then washed with PBS three times. The substrate solution was added and incubated for 30 min, where the reaction was terminated upon adding the stop solution. The absorbance was recorded at 450 nm using a microplate reader. All ELISA experiments were repeated three times.

### 2.6. Surgical Procedure and Induction of SCI

**Figure 1:**
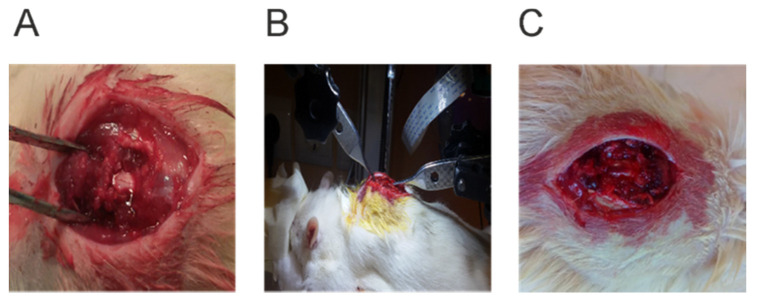
(**A**) Laminectomy of the spinal cord, (**B**) using the Horizons Impactor device, (**C**) after spinal cord injury and the formation of a hematoma.

To induce the SCI model, rats were anesthetized by inhaling the isoflurane prior to the surgery. First, rats were subject to isoflurane (5%) and oxygen (1 L/min) in a closed space, and, to maintain the anesthesia, animals were transferred to the surgical site and received a proper amount of isoflurane vapor inhalation (3–5%) and oxygen (0.8–1 L/min) from the mask until the end of operation. To begin the surgery operation, paravertebral muscles were separated on either side of the segments. To stabilize for bilateral injury, the transverse process of the upper and lower vertebra in the spine was fixed by a clamp. Next, laminectomy was performed at T10 level, and all the spinal cord-exposed rats received a force of 150 kilodynes (moderate SCI) by using the Infinite Horizons Impactor (Figure 1A–C). After SCI, the incised tissue in the wound site was closed with sutures (muscles, subcutaneous tissues, and skin), and animals were transferred under a warm lamp for 2 h in a single cage. In order to relieve pain and avoid infection, all animals received buprenorphine (0.1 mg/kg) every 6–12 h for three days and ciprofloxacin (350 mL units/days) through IP for one week, respectively. Finally, the bladder sac was discharged manually twice a day for one week.

### 2.7. Injection of MSCs-CM

In the present study, we applied two routes for delivery of MSCs-CM into the spinal cord: the focal and the IP. In the focal delivery route, 5 µL of CM was injected focally into the proximal, central, and distal parts of the lesion site immediately after SCI, and, in the IP delivery form, 500 µL CM was infused once a day via the intraperitoneal (IP) for one week.

### 2.8. Transplantation of the hAF-MSCs

MSCs were detached using 0.25% Trypsin-EDTA and were washed by PBS for three times. Following SCI, 5 × 10^5^ cells were re-suspended in 5 µL PBS and injected into the proximal, central, and distal parts of the lesion site using a capillary glass needle through a Hamilton syringe. For immunosuppression, two days before cells transplantation, all rats received cyclosporine (1 mg/100 g body weight) until the end of the test.

### 2.9. Immunofluorescence Staining

All rats were sacrificed two weeks after treatment using ketamine (100 mg/kg) and xylazine (5 mg/kg) overdose; afterward, animals were perfused, first by normal saline (NaCl 9%), then 4% formaldehyde via the left ventricle. Spinal cords were dissected out correctly and fixed with 4% formalin solution. For histological examination, spinal cord samples were dehydrated within a series of alcohols, as cleared by incubations in xylene, and were finally embedded in paraffin. Then, 4 µm-thick sections were prepared by microtome, which were mounted on poly-lysine-coated slides, dried overnight at 4 °C, following deparaffinization and rehydration in decreasing ethanol concentration and washing in tap water, and then were stored at 4 °C until use. Having been washed in phosphate-buffered saline (PBS; 0.1M, pH 7.4, and 0.9% NaCl), the antigen retrieval was carried out through the incubation of the sections in preheated 10mM sodium citrate buffer for 15 min at 100 °C. The blocking endogenous peroxidase step was also performed via the incubation of the sections in 0.6% H_2_O_2_ in PBS for 30 min. Then, the sections were exposed to the primary antibodies, anti-doublecortin antibody (ab18723) and anti-*GFAP* antibody [2A5] (ab 4648), overnight at 4 °C. Being washed with PBS for 3 times, the sections were incubated with goat anti-mouse IgG H&L (Phycoerythrin) (ab 97024) and goat anti-rabbit IgG H&L (FITC) (ab 6717) at room temperature for 1 h. Nuclei were counterstained with 4′,6-diamidino-2-phenylindole (DAPI) (ab 104139). Overall, 6 serial sections with identical intervals were selected and 5 subfields were randomly considered to IF staining. The percentages of the *DCX*- and *GFAP*-positive cells were recorded. Images were taken and observed by an Olympus fluorescence microscope, and the data were analyzed using the Image J program software plugin.

### 2.10. Statistical Analysis

The results were presented as mean ± SEM. One-way analysis of variance (ANOVA) and post hoc Tukey tests were performed to detect the statistically significant difference between groups. *p* < 0.05 was considered statistically significant. In this article, Graph Pad Prism (version 6.01; Graph Pad Software, San Diego, CA, USA) was used to analyze and depict the data.

### 2.11. Meta-Analysis and Network Analysis of DCX Expression

Meta-analysis of publicly available expression data, based on mutual ranking (MR) index, was performed to find the commonly coexpressed genes with *DCX* using *COXPRESdb v7* [21], as previously described [22]. Ranking of correlation coefficient is a robust and effective approach in meta-analysis. This method removes the batch effects between the experiments, uncovering the biological significance of gene coexpression using a considerable amount of transcriptomic data deposited in publicly available data repositories, such as *NCBI-GEO* [23]. In addition to coexpression analysis, text (literature) mining-based network analysis was performed using Pathway Studio Web application tool (Elsevier) [24], as previously described [25,26]. To this end, natural language processing (NLP) [27] was used to find the relationships between *DCX* and other proteins from biomedical texts. The relationship types, such as binding, expression, regulation, and biomarker, as well as the sentence of the paper mentioning the relationship and paper information, were recorded.

## 3. Results

### 3.1. hAF-MSCs Phenotype Identification

**Figure 2:**
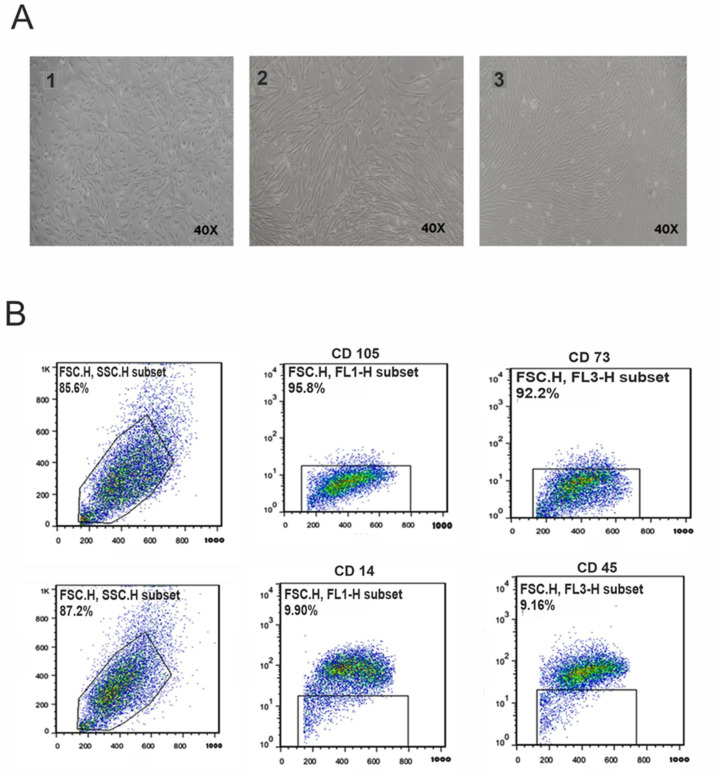
Cultivation and characterization of MSCs. (**A**) 1 shows P0 of cells after one week of the first culture; 2 shows P1 of cells after three days; 3 shows P1 of cells after one week (40× magnification was used). (**B**) Flow cytometry of hAF-MSCs for surface markers. Expression of surface markers detected by flow cytometric analysis in P4 of MSCs, with CD73 and CD105 as positive markers and CD14 and CD45 as negative markers for hAF-MSCs.

Bright-field microscopic observations showed that the MSCs had a typical spindle-shaped or fibroblast-like morphology in the (P3–P4) cell culture passages (Figure 2A). Flow cytometry analysis revealed that the MSCs were positive for the MSC markers CD105 (95.80%) and CD73 (92.2%) and failed to express the hematopoietic markers CD14 (9.90%) and CD45 (9.16%) (Figure 2B).

### 3.2. ELISA Analysis

ELISA analysis was performed to evaluate the secretory potential of hAF-MSCs. Our results reveal that the amount of BDNF, IL-1β, and IL-6 proteins in the given CM, were 24 ± 0/375, 12 ± 0/73, and 10 ± 0/562 pg/mL, respectively (Table 1).

### 3.3. hAF-MSCs Promote the Neuroblast Expression as an Indicator of Neurogenesis

**Figure 3:**
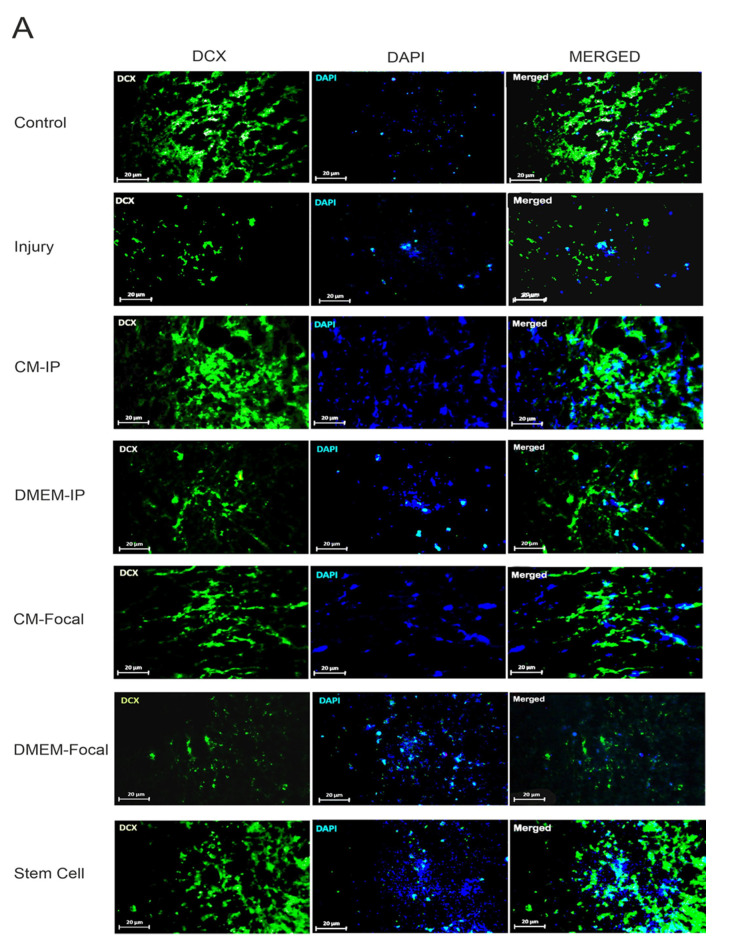

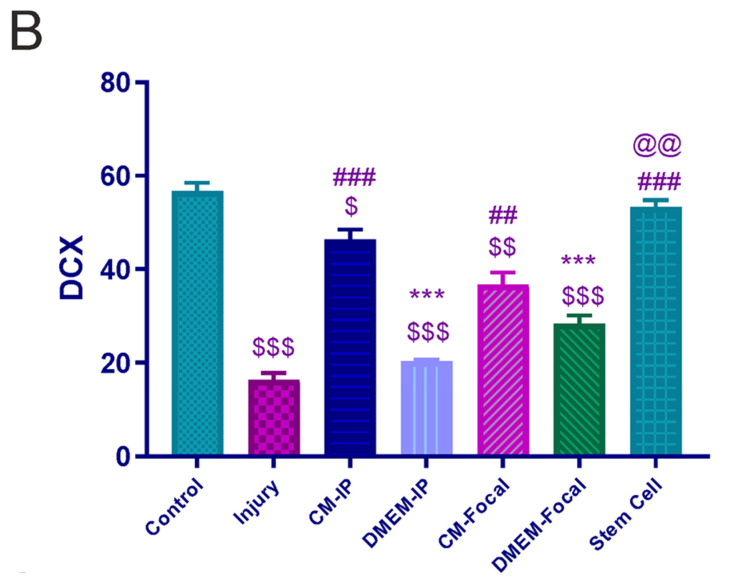
Immunofluorescence staining two weeks’ post-injury for *DCX^+^*. (**A**) The double staining of *DCX* for the effect of hAFMSC-CM on *DCX* in spinal cord injury by confocal microscopy. *DCX*-positive neuroblasts (green, 2 weeks’ post-injury) were detected in the spinal cord. Nuclei were counterstained with DAPI(blue). After SCI treatment with CM in the form of IP and Focal, stem cells showed an increase in *DCX* (*p* < 0.001). Data represent means ± SEM. Scale bars = 20 µm. (**B**), the quantification of immunostaining data showed an increase in *DCX^+^* cells in different groups. $$$ *p* < 0.001, $$ *p* < 0.01, $ *p* < 0.05 versus control. ### *p* < 0.001, ## *p* < 0.01, versus injury. *** *p* < 0.001, versus CM-IP. @@ *p* < 0.01, versus CM-Focal.

To explore the impact of hAF-MSCs and MSCs-CM on the neuroblast expression at the protein level, the immunofluorescence staining was used (Figure 3A,B). Our results show a significant difference in the number of *DCX*-positive cells among groups. Within the immunofluorescence evaluation section, statistical analysis indicated a significant difference in the Stem Cell group relative to the injury group (*p* < 0.001) and the CM-Focal group (*p* < 0.01). In addition, our data revealed that the IP and focal delivery routes of MSCs-CM significantly increased the number of *DCX*-positive cells relative to the injury group (*p* < 0.001, *p* < 0.01), respectively. Moreover, the number of *DCX*-positive cells failed to increase in the DMEM groups relative to the injury group (*p* > 0.05). Collectively, these data showed that although the CM, particularly through the IP delivery route, could promote the neurogenesis at a partial level, stem cells have superior effects on the neurogenesis enhancement relative to CM alone. Thus, the juxtacrine activity of stem cells could be more effective than their paracrine mechanism.

### 3.4. hAF-MSCs Suppress the Astrogliosis and Glial Scar

**Figure 4:**
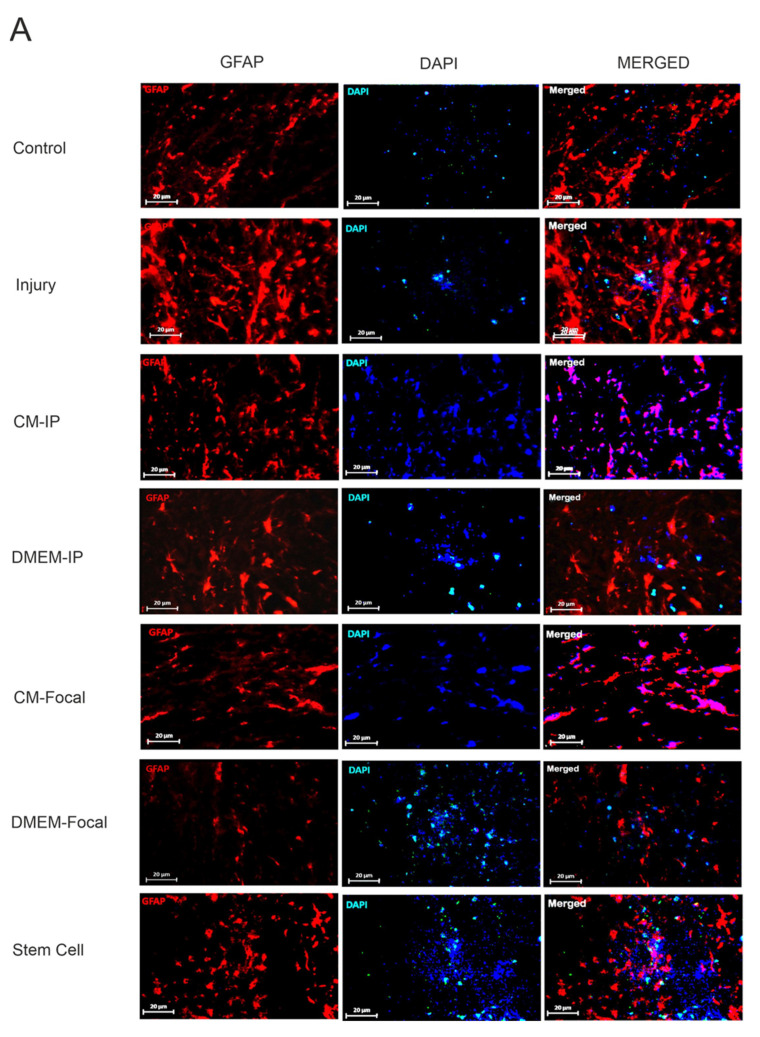

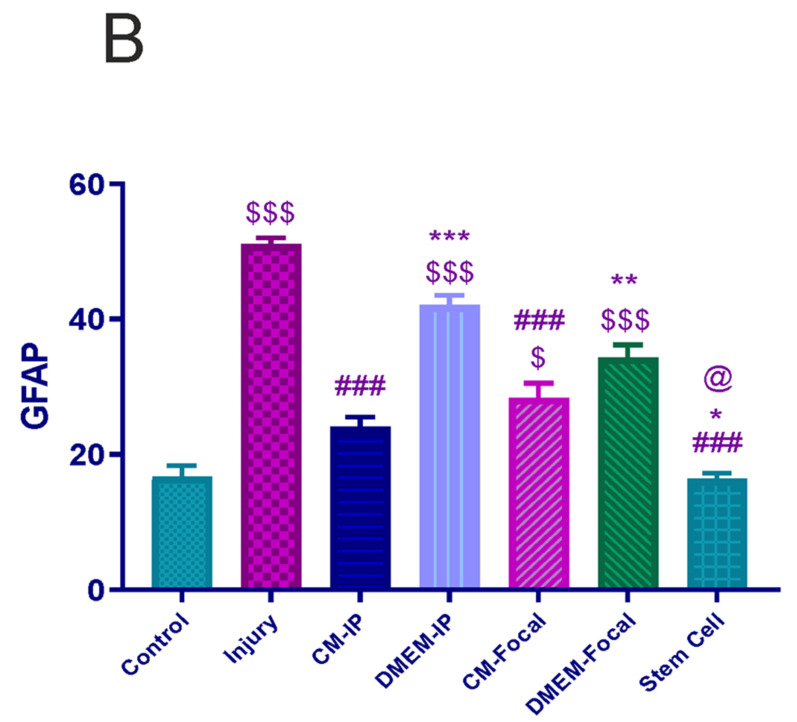
Immunofluorescence staining within two weeks’ post-injury for *GFAP*. (**A**) The double staining of *GFAP* for the effect of hAFMSCs-CM on *GFAP* in spinal cord injury by confocal microscopy. *GFAP*-positive astrocytes (red, 2 weeks’ post-injury) is detected in the spinal cord. Nuclei were counterstained with DAPI (blue). After SCI treatment with CM in the form of IP and Focal, the stem cells showed a significant decrease in *GFAP* (*p* < 0.001). Data represent means ± SEM. Scale bars = 20 µm. (**B**) The quantification of immunostaining data showed a decrease in *GFAP^+^* cells within different groups. $$$ *p* < 0.001, versus control. ### *p* < 0.001, versus injury. *** *p* < 0.001, ** *p* < 0.01, * *p* < 0.05, versus CM-IP. @ *p* < 0.05 versus CM-Focal.

To examine whether MSCs and their CM could affect astrocyte proliferation and astrogliosis, we evaluated the expression level of *GFAP* protein using immunofluorescence staining (Figure 4A,B). Our data from the immunofluorescence imaging indicated a significant difference in the number of *GFAP*-positive cells among groups. Statistical analysis showed that the Stem Cell group had a significantly decreased number of astrocytes relative to the injury group (*p* < 0.001), CM-IP (*p* < 0.05), and CM-Focal (*p* < 0.05) groups. Our data revealed that the CM-IP and CM-Focal groups significantly decreased the number of *GFAP*-positive cells relative to the injury group (*p* < 0.001). Moreover, the presented results demonstrate a significant difference in the *GFAP* protein level in the CM-IP and CM-Focal groups relative to the injury group (*p* < 0.001) (see Figure 4B). Taken together, MSCs could significantly suppress the astrogliosis associated with the other groups, and the CM infusion, via the IP, was found to be more effective than the focal injection.

### 3.5. Common Coexpressed Genes with DCX and Its Gene Interaction Network

**Figure 5:**
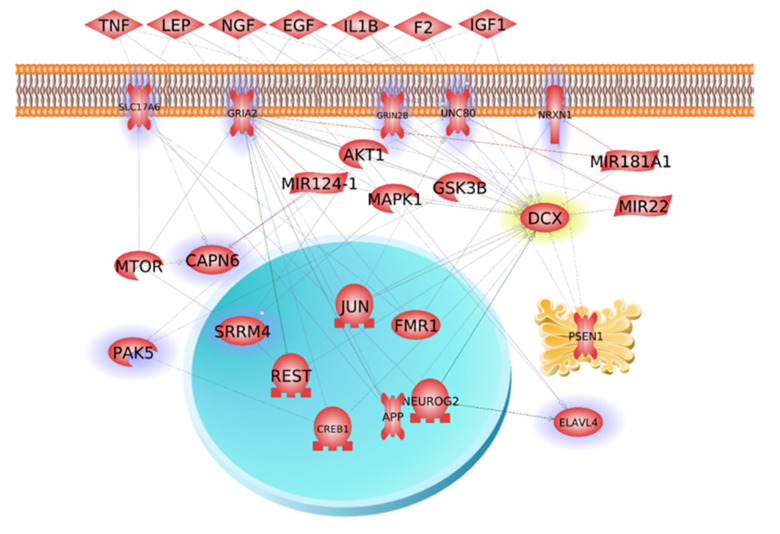
Gene interaction network analysis of *DCX.* Genes with high level of positive coexpression with *DCX*, based on meta-analysis, are highlighted.

Table 2 illustrates the top 10 positively coexpressed genes with *DCX* and their correlations by meta-analysis of transcriptomic data. Figure 5 demonstrates the gene interaction network of *DCX. KEGG* enrichment analysis showed that these co-expressed genes are involved in axon guidance, retrograde endocannabinoid signaling, and glutamatergic synapse. Notably, *PAK5*, *ST8SIA3*, and *NRXN1* are involved in neuroactive ligand–receptor interaction

## 4. Discussion

In the current study, the restorative potential of the MSCs and their CM in the rat model of SCI was compared in terms of the cellular and molecular approaches. In this research, the MSCs were transplanted in the lesion site on the dorsal funiculus at the T10 level of the vertebral column, and the MSCs-CM were injected through the intraperitoneal and focal delivery routes. Our data from the immunofluorescence experiment revealed that the expression level of *DCX* was enhanced within the MSCs and CM-treated groups relative to the untreated groups. Noteworthily, the MSCs significantly increased the level of *DCX* expression in comparison to CM following the two weeks of SCI. Doublecortin (*DCX*) is a microtubule-associated protein expressed by immature neurons in embryonic and adult cortical structures, which is considered as an indicator of neurogenesis markers [28,29]. The results from the *GFAP* IF staining unveil that the MSCs could increase the level of this protein more than that of the CM. It should be noted that the CM injection through IP could significantly decrease the expression of GFAP relative to the focal administration route. These findings demonstrate that the MSCs and CM stimulated the neuroblast proliferation as well as the neurogenesis and decreased the astrocyte proliferation and astrogliosis. Another hallmark result in our research was the evaluation of the efficacy of the administration route on astrocytosis. Notably, our data revealed that the IP injection of CM could decrease the astrocytosis phenomenon as compared with the focal injection group. Generally, neurogenesis occurred at a low level within the adults’ spinal cord under normal conditions. However, in some pathologic conditions, such as SCI, the endogenous neurogenesis increased, to some extent, to replace neural loss, yet this compensation was not satisfactory [30]. A great deal of research has revealed that the stem cells and neurotrophic factors increased neurogenesis and improved the functional behavior in the SCI [31]. Additionally, other studies showed that cell transplantation could replace the lost cells and rescue the functional deficits in the SCI; however, some of the transplanted cells died due to an undesirable microenvironment, glial scar, lack of trophic factor, and oxidative stress [32]. Kaneko et al. showed that the stem cells transplantation through focal (directly) or CSF (indirectly), within 1–3 weeks, led to axonal regeneration and functional recovery in SCI [33,34]. For the first time, we showed that cell therapy using MSCs, immediately after spinal cord injury (focal route), has a positive effect on SCI treatment. We observed that the MSCs following the SCI improved the proliferation of neuroblast cells and suppressed the astrocytosis, similarly to the control group. It is likely that the transplanted cells are transdifferentiated into neuroblasts and the immature neurons, where it integrated into the host spinal cord tissue, leading to tissue repair and functional improvement [35,36,37]. Despite several studies, the differentiation of mesenchymal stem cells into the neuron and glial cells is controversial [8]. There is no solid evidence that transplanted stem cells remain intact and unchanged for a long time after transplantation [30]. So far, no effective methods have been proposed to control the final fate of transplanted stem cells to appropriately differentiate, proliferate, and integrate into the new tissue [30]. Therapeutic effects of MSCs transplantation could be due to the secretion of the neurotrophic factors and their anti-apoptotic and anti-inflammatory activities. In this regard, we decided to examine the existence of some neurotrophic factors, mainly the BDNF, anti-apoptotic, and anti-inflammatory cytokines, including Il-6 and Il-1 β in the CM of the MSCs. Our ELISA experiment showed that the MSCs could release the mentioned factors in their CM. Based on the well-documented study, it has been revealed that neurotrophic factors are indispensable for axonal outgrowth, tissue repair, and functional developments [30,36]. BDNF is a type of neurotrophin that affects SCI treatment in different ways, working as a trophic support by enhancing neural cell survival and axonal regeneration [38]. It has been shown that the IL-6 has the capacity to suppress inflammatory responses and provide a desirable microenvironment for neural regeneration and repair in acute CNS injuries. [39]. Previously, the significance of MSC-CM was demonstrated via the focal, intravenous, lateral ventricle intravenous, and intrathecal injection in the repair of CNS [19,30,39,40,41]. Our study showed that the infusion of the CM through IP for seven days could promote neurogenesis and inhibit astrocytosis. This could be attributed to the repeated injection of CM throughout the 7 days, which increased the factors stimulating the intrinsic ability of regeneration following SCI. In a similar study, Dorothee et al. demonstrated that the use of BMSC-CM in the lesion within the SCI is beneficial and not harmful [19]. In addition, the injection of CM can promote locomotion recovery after six weeks and reduce the lesion size; however, it failed to increase axonal regrowth [19,20]. In line with our study, Zhang et al. demonstrated that the MSCs can produce many kinds of trophic factors both in vitro and in vivo and promote neural regeneration [42,43]. Furthermore, we found a high correspondence between meta-analysis of the co-expression data of *DCX* and literature mining-based network analysis. *PAK5*, *ST8SIA3*, and *NRXN1* could positively coexpress with *DCX* and played a role in the neuroactive ligand–receptor interaction. The present study indicated that hAF-MSCs transplantation, as compared with the resulting supernatant, could be more effective in neurogenesis induction and astrocytosis inhibition. Noteworthily, the present work had some limitations. In this research, we did not examine the trace and fate of the transplanted MSCs and their functional integration with the host tissue.

## 5. Conclusions

In the present study, we investigated the effective potential of hAF-MSCs and their CM in the SCI model. Our results show that the MSCs enhanced the neurogenesis and suppressed the astrogliosis. Moreover, the administration route of the CM into the spinal cord was found to be a critical issue in the regenerative approaches. The delivery of the CM via IP route relative to the focal injection significantly suppressed astrogliosis.

## Figures and Tables

**Table 1:** The measurement of BDNF, IL-1β, IL-6 in CM.

BDNF	IL-1β	IL-6
24 ± 0/375 pg/mL	12 ± 0/73 pg/mL	10 ± 0/562 pg/mL

**Table 2:** Top 10 coexpressed genes with DCX revealed by meta-analysis of publicly available transcriptomic data.

Genes	Name	Meta-Analysis Based Correlation (%)	Number of Experiments Used for Identifying Correlation
*PAK5*	p21 (RAC1) activated kinase 5°	28.7%	20
*ELAVL4*	ELAV like RNA binding protein 4	43.7%	20
*CAPN6*	calpain 6	19.3%	22
*NRXN1*	neurexin 1°	34.0%	28
*ST8SIA3*	°ST8 alpha-N-acetyl-neuraminide alpha-2,8-sialyltransferase 3	33.0%	35
*NOL4*	nucleolar protein 4	30.7%	22
*GRIA2*	glutamate ionotropic receptor AMPA type subunit 2	28.9%	16
*UNC80*	unc-80 homolog, NALCN channel complex subunit	33.3%	21
*SRRM4*	serine/arginine repetitive matrix 4	32.6%	31
*SLC17A6*	solute carrier family 17 member 6°	30.2%	20

## Data Availability

Data will be delivered on request and corresponding author is responsible to respond any questions regarding the originality of data.
